# Endogenous Retrovirus Elements Are Co-Expressed with IFN Stimulation Genes in the JAK–STAT Pathway

**DOI:** 10.3390/v15010060

**Published:** 2022-12-24

**Authors:** Yanglan Wang, Mengying Liu, Xing Guo, Bohan Zhang, Hanping Li, Yongjian Liu, Jingwan Han, Lei Jia, Lin Li

**Affiliations:** 1 College of Life Science and Technology, Beijing University of Chemical Technology, Beijing 100029, China; 2 Department of Virology, Beijing Institute of Microbiology and Epidemiology, Beijing 100071, China; 3 State Key Laboratory of Pathogen and Biosecurity, Beijing 100071, China; 4 Department of Microbiology, School of Basic Medicine, Anhui Medical University, Hefei 230032, China

**Keywords:** IFN-β, ERV, LTR, DEHERV-G pairs, ISGs

## Abstract

**Background**: Endogenous retrovirus (ERV) elements can act as proximal regulatory elements in promoting interferon (IFN) responses. Previous relevant studies have mainly focused on IFN-stimulated genes (ISGs). However, the role of ERV elements as cis-regulatory motifs in regulating genes of the JAK–STAT pathway remains poorly understood. In our study, we analyzed the changes in ERV elements and genes under both IFN stimulation and blockade of the signaling pathway. **Methods**: The effects of interferon on cells under normal conditions and knockout of the receptor were compared based on the THP1_IFNAR1_KO and THP1_IFNAR2_mutant cell lines. The correlation between differentially expressed ERVs (DHERVs) and differentially expressed genes (DEGs) as DEHERV-G pairs was explored with construction of gene regulatory networks related to ERV and induced by proinflammatory cytokines. **Results**: A total of 430 DEHERV loci and 190 DEGs were identified in 842 DEHERV-G pairs that are common to the three groups. More than 87% of DEHERV-G pairs demonstrated a consistent expression pattern. ISGs such as *AIM2*, *IFIT1*, *IFIT2*, *IFIT3*, *STAT1*, and *IRF* were activated via the JAK–STAT pathway in response to interferon stimulation. Thus, *STAT1*, *STAT2*, and *IRF1* appear to play core roles in regulatory networks and are closely associated with ERVs. **Conclusions**: The RNA expression of ISGs and ERV elements is correlated, indicating that ERV elements are closely linked to host innate immune responses.

## 1. Introduction

Endogenous retroviruses (ERVs) entered the germ line of our ancestors over millions of years of evolution; they then spread vertically in a Mendelian manner and remained stable in the human genome, where it is estimated that they account for about 8% of the genome [1,2]. ERVs are retrotransposons (REs), a type of transposable element (TE) that moves by a ‘copy and paste’ mechanism involving the reverse transcription of an RNA intermediate and insertion of its cDNA copy into a new position within the host genome [3,4,5]. ERVs are structurally similar to the proviruses of common retroviruses, where the *gag*, *pol*, and *env* genes are flanked by two long terminal repeats (LTRs) [6]. Most ERVs exist in the form of solo LTRs produced by homologous recombination between the 5′ and 3′ LTRs [3,7]. These LTR elements were shown to influence gene regulation by providing cis-regulatory motifs and attracting DNA and histone-modifying complexes [3,7,8,9,10]. 

Double-stranded RNA (dsRNA) stress caused by ERVs is sensed through endosomal toll-like receptor 3 (TLR3), retinoic-acid-inducible gene I (RIG-I), or melanoma differentiation-associated gene 5 (MDA5), leading to the expression of the type I IFN, IFN-β [11,12,13]. The type I interferon receptor (IFNAR) is composed of transmembrane heterodimers IFNAR1 and IFNAR2. IFN-β interacts with type I interferon receptors to induce the classical JAK–STAT signaling pathway [11,14,15].

The first step in IFN-mediated signaling is to activate JAK1 and TYK2, members of the receptor-associated Janus-activated kinase (JAK) family, through IFN-β binding to IFNAR. Activation of JAK then leads to tyrosine phosphorylation of STAT2 (signal transducer and activator of transcription 2) and STAT1, with formation of STAT1–STAT2 dimers or STAT1–STAT1 homodimers. In step three, IFN-regulatory factor 9 (IRF9) combines with STAT1–STAT2 complexes, resulting in the formation of STAT1–STAT2–IRF9 complexes, which are known as IFN-stimulated gene factor 3 (ISGF3) complexes. Finally, ISGF3 or STAT1–STAT1 homodimers enter the nucleus and act on the interferon-stimulated regulatory element (ISRE) or IFN-γ-activated site (GAS) elements on the promoter of the IFN stimulation genes (ISGs) to initiate transcription of these genes [14,15]. 

ERV elements control various aspects of the interferon response. In particular, a member of the ERV family is often co-opted as the proximal enhancer to promote interferon responses [16,17,18]. Members of the primate-specific MER41 family harbor binding motifs for the transcription factors IRF1 (interferon regulatory factor 1) and STAT1 [18,19]. Following IFNG induction, copies of MER41 bind to STAT1 and IRF1 and enhance activity. *AIM2* encodes an interferon-stimulated protein that detects dsDNA and triggers an inflammatory response when viral or bacterial infection occurs. *AIM2* is immediately adjacent to a MER41 element, and MER41 deletion prevents *AIM2* expression after IFNG induction and thus reduces the downstream inflammatory response [18,19].

Type I IFN promotes a change in the expression of over 1000 genes, leading to a wide variety of cellular responses [20]. A great deal of work has been invested in understanding the complexity of type I interferon signaling. However, it is present in many different cell types and organisms and may show different responses [21,22]. In our study, we analyzed the changes in ERV elements and genes under both IFN-β stimulation and blockade of the signaling pathway based on the THP1_IFNAR1_KO and THP1_IFNAR2_mutant cell lines. Our analysis shows a correlation between the expression of ISGs and ERVs when their genomic distance is within 100 kb. Characterizing the relationship between ISGs and ERV elements in terms of position and expression will help us in the future to study the regulatory role of ERV elements on ISGs in IFN signal transduction.

## 2. Materials and Method

### 2.1. RNA-Seq Data Acquisition

The data in this publication were deposited in the NCBI Gene Expression Omnibus [23,24] and are accessible through GEO Series accession number GSE211502. In brief, IFNAR1 knockout and IFNAR2 mutant THP1 cell lines were established using the CRISPR–Cas9 method [25,26,27]. The mutation was introduced in exon 2 and 3 of IFNAR1 and IFNAR2, respectively. Cells were treated with a control medium or 1 ng/mL IFN-β for three hours. Two duplicates were tested for each treatment. 

### 2.2. Reads Mapping and Counting

In general, FastQC (v 0.11.5, Babraham Bioinformatics, Cambridge, England) [28] was used for the quality control of raw data, and Trimmomatic (v 0.38, USADEL LAB, Dusseldorf, Germany) was used to filter out lower-quality sequences [29]. The filtered sequences were aligned to the human reference genome GRCh38 using the HISAT2 (v 2.1.0, Center for Computational Biology, Maryland, USA) [30] tool and counted using the “primary” option of featureCounts (v 1.6.3, Bioinformatics Division, Melbourne, Australia) [31]. Genes were annotated using Ensembl GRCh38 version 95 GTF, whereas GTF-annotated genes were from the HERV database, which contains the most extensive information on HERV loci for HERV locus annotation [32].

### 2.3. Differential Expression Analysis

The DESeq2 package (v1.38.2, Bioconductor, New York, NY, USA) was used to perform differential expression analysis of HERV loci and human genes [33]. Genes with *p* < 0.05 and |log2 fold change| > 1 were considered to be significantly differentially expressed [34]. When both HERV and its neighboring human genes were differentially expressed (DEHERVs and DEGs), we considered them as DEHERV-G pairs and determined the number of DEHERV-G pairs in each dataset. Venn diagrams prepared using Weishengxin online software (http://www.bioinformatics.com.cn, access date: 28 April 2022) to show the overlap in different groups. RIdeogram (v.1.0, Laboratory of Biochemistry, Wageningen, Netherlands) was used to show the location of HERVs and genes on the chromosomes.

### 2.4. GO and KEGG-Enrichment Analyses

The Database for Annotation, Visualization, and Integrated Discovery (DAVID, http://david.ncifcrf.gov, version 6.8, access date: 9 May 2022) [35] is an online biological information database that integrates biological data and analysis tools and provides a comprehensive set of functional annotation information of genes and proteins for users to extract biological information. Gene Ontology (GO) is a major bioinformatics tool to annotate genes and analyze the biological process of these genes [36]. Kyoto Encyclopedia of Genes and Genomes (KEGG) is a database resource for understanding high-level functions and biological systems from large-scale molecular datasets generated by high-throughput experimental technologies [37]. To analyze the function of key proteins, biological analyses were performed using the DAVID online database. *p* < 0.05 was considered statistically significant.

### 2.5. Construction of Protein–Protein Interaction (PPI) Networks

Analyzing functional interactions enables a better understanding of the correlation between proteins in different pathways. The PPI network was predicted using the Search Tool for the Retrieval of Interacting Genes (STRING; http://string-db.org, access date: 16 May 2022) online database [38]. In our study, PPI networks of key proteins were constructed using the STRING database, with associations being considered statistically significant for combined score > 0.4. The PPI networks were drawn using Cytoscape (v 3.7.2, NIGMS, Bethesda, USA), and the most significant modules in the PPI networks were identified using MCODE algorithm [39]. The criteria for selection were as follows: degree cut-off = 2, node score cut-off = 0.2, max depth = 100, and k-score = 2.

## 3. Results

### 3.1. IFNAR Knockout Validation

THP1 cell lines with the IFNAR1 gene knocked out (THP1_IFNAR1_KO cells) and THP1 cell lines with residual protein expression due to exon skipping of IFNAR2 (THP1_IFNAR2_mutant cells) were obtained from Pr. Liguo Zhang as gifts [27]. The knockout cell line has a 4 bp deletion in one allele of IFNAR1 and a 1 bp insertion in the other allele; the THP1 cell clone has a 7 bp deletion in both IFNAR2 alleles. The membrane expression of IFNAR1 and IFNAR2 was shown to be decreased based on the level of staining using antibodies against the specific isotype of interest in THP1_IFNAR1_KO cells and THP1_IFNAR2_mutant cells, respectively.

### 3.2. RNA-Seq Datasets Analysis

**Figure 1:**
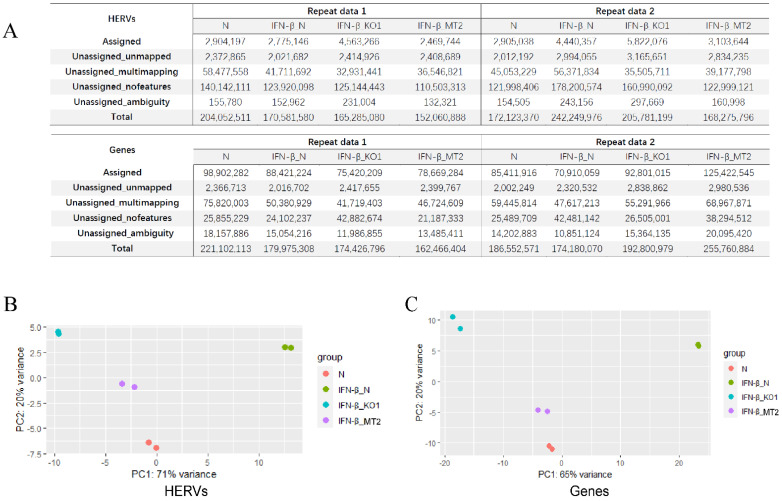
Clean reads acquired and PCA analysis. (**A**) Clean reads of HERVs and genes mapped on the reference genome, including four datasets with two duplicates: IFN-β-treated THP1 cells (IFN-β_N), IFN-β-treated THP1_IFNAR1_KO cells (IFN-β_KO1), IFN-β-treated THP1_IFNAR2_mutant cells (IFN-β_MT2), and the untreated THP1 cells (N). PCA analysis of the transformed expression values of HERV loci (**B**) and human genes (**C**).

To study the contribution of the ERV family to IFN signaling, a total of four datasets (two duplicates) including the IFN-β-treated THP1 cells (IFN-β_N), IFN-β-treated THP1_IFNAR1_KO cells (IFN-β_KO1), IFN-β-treated THP1_IFNAR2_mutant cells (IFN-β_MT2), and untreated THP1 cells (N) were used for analysis. After quality control of the raw reads, we obtained a total of about 50 G of clean reads. After annotation by Ensembl GRCh38 and repeatMasker, we detected the expression of HERVs and human genes in all analyzed samples. Clean reads of HERVs and genes were compared against sequences of the reference genome (Figure 1A). To eliminate background interference, RNA-seq analysis of the untreated THP1_IFNAR1_KO cells and the untreated THP1_IFNAR2_mutant cells was also performed. The clean reads of HERVs and genes are shown in Supplementary Table S1. 

Principal component analysis (PCA) of the expression of HERVs and genes from the four datasets with two duplicates are shown in Figure 1B,C. The samples cluster within a group and are dispersed among the groups. The results show there are distinct expression patterns of HERVs and genes across the four datasets. 

### 3.3. Screening and Classification of DEHERVs 

These datasets were divided into three groups: KO1 (IFN-β-treated THP1 cells vs. IFN-β-treated THP1_IFNAR1_KO cells), KO2 (IFN-β-treated THP1 cells vs. IFN-β-treated THP1_IFNAR2_mutant cells), and N (IFN-β-treated THP1 cells vs. untreated THP1 cells). We used the KO1 and KO2 groups to analyze the changes in HERVs after knockout of the IFNAR1 or mutant of the IFNAR2 under interferon stimulation conditions; we also analyzed the changes in HERVs under interferon stimulation versus no interferon stimulation with group N. To eliminate background interference, we analyzed HERVs expression in different situations, including the changes in HERVs in THP1_IFNAR1_KO cell lines with and without interferon stimulation (group N1); the changes in HERVs in THP1_IFNAR2_mutant cell lines with or without interferon stimulation (group N2); the changes in HERVs between THP1 and THP1_IFNAR1_KO cell lines without interferon stimulation (group N3); and the changes in HERVs between THP1 and THP1_IFNAR2_mutant cell lines without interferon stimulation (group N4).

**Figure 2:**
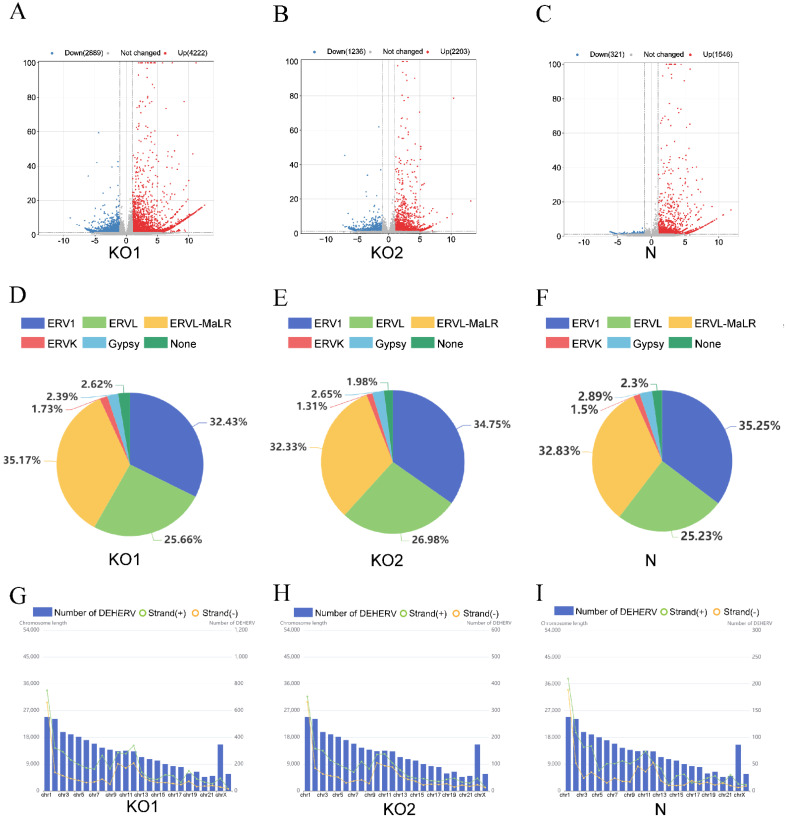
Screening and classification of DEHERVs. (**A**–**C**) Volcano plots of the three groups (KO1, KO2, and N). Red and blue-colored dots represent upregulated and downregulated HERV loci, respectively (adjusted P-value < 0.05 and |log2 fold change| > 1). Gray-colored dots represent loci with no significant differential expression. (**D**–**F**) Pie charts showing the number of DEHERVs in each superfamily of three groups. (**G**–**I**) Distribution of DEHERV loci on the plus (green point) and minus (orange point) strands of each chromosome. The blue bar represents the length of each human chromosome.

We obtained three datasets according to the screening conditions *p* < 0.05 and |log2 fold change| > 1. Under IFN-β stimulation, IFNAR1 knockout resulted in 2889 downregulated loci and 4222 upregulated loci, and IFNAR2 mutant resulted in 1236 downregulated loci and 2203 upregulated loci. In normal THP1 cells, differential expression analysis showed 1546 upregulated loci and 321 downregulated loci with IFN-β stimulation (Figure 2A–C). When IFNAR1 or IFNAR2 is knocked out, interferon stimulation causes few changes in HERVs (Supplementary Figure 1A,B). In addition, we analyzed the changes in HERVs caused by interferon production in the intracellular environment in the absence of interferon stimulation (Supplementary Figure S1C,D).

To explore whether IFN-β causes changes in HERV expression, we analyzed the characteristics of the location and classification of the DEHERVs. According to the Dfam database [40], DEHERVs can be classified into six superfamilies (ERV1, ERVL, ERVK, Gypsy, ERVL-MaLR, and unclassified). In the three groups of DEHERVs, approximately one-third of DEHERVs belong to the ERVL-MaLR superfamily, one-third to the ERV1 superfamily, one-quarter to the ERVL superfamily, and only 1.5% to the ERVK superfamily (Figure 2D–F).

The DEHERVs of the three groups are widely distributed in the human genome, and the number of DEHERVs on each chromosome (except on the X chromosome) is positively correlated with chromosome size. In addition, there are significantly more DEHERVs on the plus strands than on the minus strands (*p* < 0.0001). In particular, 4540 loci (63.8%) are found located on the plus strands and 2571 loci (36.2%) on the minus strands of the KO1 group; 2109 loci (61.3%) are located on the plus strands and 1330 loci (38.7%) on the minus strands of the KO2 group; 1184 loci (63.4%) are located on the plus strands and 683 loci (36.6%) on the minus strands of the N group (Figure 2G–I).

### 3.4. Identification and Functional Enrichment of DEGs

**Figure 3:**
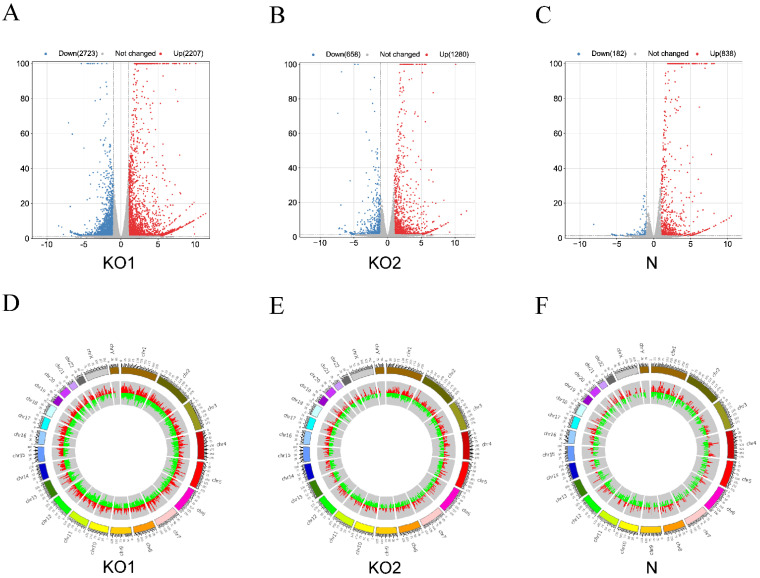
Screening and classification of differentially expressed genes (DEGs). (**A**–**C**) Volcano plots of the three groups (KO1, KO2, and N). Red- and blue-colored dots represent upregulated and downregulated genes, respectively (adjusted P-value < 0.05 and |log2 fold change| > 1). Gray-colored dots represent loci with no significant differential expression. (**D**–**F**) Circos plots showing DEGs (red column) and DEHERVs (green column) across chromosomal locations. The length of the column represents the absolute value of the log2 fold change.

We further analyzed the differences in the cellular gene expression level of the three groups according to the screening conditions in which there is *p* < 0.05 and |log2 fold change| > 1. Under IFN-β stimulation, IFNAR1 knockout resulted in 2723 downregulated genes and 2207 upregulated genes, and IFNAR2 knockout resulted in 658 downregulated loci and 1280 upregulated loci. In THP1 cells, differential expression analysis showed 838 upregulated genes and 182 downregulated loci with IFN-β stimulation (Figure 3A–C). Differentially expressed genes were found throughout the genome, with patterns similar to those observed for HERVs, revealing that the expression of DEHERVs and DEGs may be correlated (Figure 3D–F). In contrast, we did not observe a consistent pattern of expression of HERVs and genes in the control group (Supplementary Figure S2A–D).

### 3.5. Identification of DEHERV-G Pairs in Each Dataset

**Figure 4:**
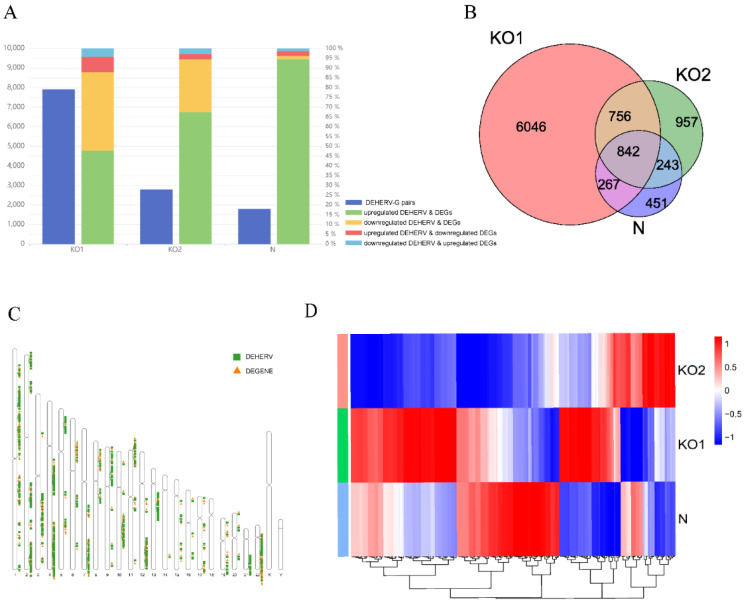
Identification of DEHERV-G pairs in each dataset. (**A**) Number and proportion of DEHERV-G pairs in the three groups. The dark blue column located on the left side of each group indicates the number of DEHERV-G pairs, and the column located on the right side of each group represents the proportion of four patterns: upregulated DEHERV and DEGs (green), downregulated DEHERV and DEGs (yellow), upregulated DEHERV and downregulated DEGs (red), and downregulated DEHERV and upregulated DEGs (light blue). (**B**) A Venn diagram of the DEHERV-G pair overlap across the three groups. Pink represents the KO1 group, green represents the KO2 group, and purple represents the N group. (**C**) Distribution of the components of the common DEHERV-G pairs on chromosomes. The green rectangle represents a DEHERV site, and the yellow triangle represents a DEG. (**D**) Heatmap of the different groups. Red indicates significantly increased gene expression, and blue indicates significantly decreased gene expression, with the intensity of color change from blue to red corresponding to a normalized log2 fold change from values −1 to 1.

To further explore the correlation between DEHERVs and DEGs in the KO1, KO2, and N groups, we defined DEHERV-G as a pair comprising HERV and its nearest human genes (within 100 kb) on the same strand with both differentially expressed [41]. The DEHERV-G pairs were divided into four patterns: upregulated DEHERV and DEGs, upregulated DEHERV and downregulated DEGs, downregulated DEHERV and DEGs, and downregulated DEHERV and upregulated DEGs. There were 7911, 2798, and 1803 DEHERV-G pairs for KO1, KO2, and N, respectively. The pattern of upregulated DEHERV and DEGs accounted for the largest proportion, with 47%, 67%, and 93% for KO1, KO2, and N (Figure 4A). More than 87% of DEHERV-G pairs in all datasets had a consistent expression pattern; that is, expression of both components was either upregulated or downregulated. As listed in Supplementary Table S2, a total of 430 differentially expressed DEHERV loci and 190 differentially expressed DEGs were identified in 842 DEHERV-G pairs common to all three groups (Figure 4B). 

In terms of distribution, these common DEHERV-G pairs are not randomly distributed but mainly concentrated on autosomes (Figure 4C). Overall, these results indicate that the expression of HERVs corresponds to that of their neighboring genes. In addition, we screened the DEGs of the three groups and found that some genes with significant differential expression (log2 fold change > 5) were ISGs, including *AIM2*, *IFIT1*, *IFIT2*, and *IFIT3* (Figure 4D, Supplementary Table S3) [42]. After stimulation, IFNs bind to their heterodimeric receptors to initiate downstream signaling cascades that lead to the upregulation of ISGs [14]. These results show that IFNAR1 and IFNAR2 are the inevitable pathways for activating these ISGs. Expression of these genes were significantly reduced after the knockout of the receptor and significantly increased in the presence of the receptor under IFN-β stimulation. 

### 3.6. Functional Enrichment Analysis of DEHERV-G Pair Genes and Protein–Protein Interaction (PPI) Network Construction

**Figure 5:**
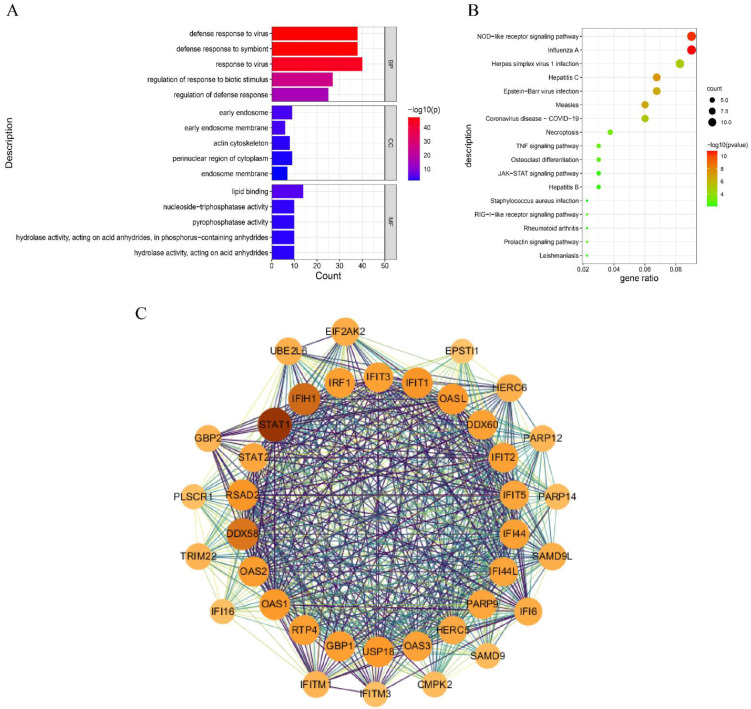
Functional enrichment analysis of DEHERV-G pair genes and PPI networks. GO analysis (**A**) and KEGG analysis (**B**) of DEGs with the top five biological processes (BPs), cell components (CC), and molecular function (MF) with the lowest *p* value (*p* < 0.05) of GO analysis included. The color of columns or bubbles represents the *p* value, and the length of the column or the size of the bubbles represents the number of counts. (**C**) PPI core networks of human genes. Circles represent protein-coding genes, and the darker the circle, the more genes are associated.

To further explore the correlation between genes of the DEHERV-G pairs, we performed GO-enrichment analysis of genes that intersected the three groups. These genes were enriched in response to viruses, defense response to viruses, defense response to symbionts, regulation of response to biotic stimulus, and regulation of defense response biological process (BP) terms (Figure 5A). Moreover, the KEGG pathway analysis showed that these genes are significantly enriched in two immune-related pathways, specifically the NOD-like receptor signaling pathway and influenza A (Figure 5B).

To identify the protein–protein interactions of these proteins encoded by DEGs, the PPI networks of key proteins that intersected the three groups are constructed. After using the MCODE algorithm to extract the core network, a total of 37 nodes, and 1433 edges were established in the PPI (Supplementary Table S4). The key genes are *STAT1*, *STAT2*, and *IRF1*, with 54, 38, and 42 linked genes, respectively (Figure 5C). 

## 4. Discussion

ISGs activated by interferon receptors play an important role in innate immune defenses. The effects of type I IFNs on the transcriptome of several cell types have been investigated in previous studies [42,43,44,45]. However, previous relevant studies have focused on ISGs, whereas the role of ERV elements as cis-regulatory motifs in regulating genes of the JAK–STAT pathway remains poorly understood. 

In our study, based on the THP1_IFNAR1_KO and THP1_IFNAR2_mutant cell lines, the effects of interferon on cells under normal conditions and knockout of the receptor were compared. We screened and explored the correlation between DEHERVs and DEGs as DEHERV-G pairs. 

Firstly, more than 87% of DEHERV-G pairs in all datasets had a consistent expression pattern, revealing correlations between ISGs and ERV elements. Secondly, a total of 430 shared DEHERV loci and 190 shared DEGs were identified in 842 DEHERV-G pairs that are common to the three groups. The ISGs in the 190 shared DEGs such as *AIM2*, *IFIT1*, *IFIT2*, *IFIT3*, *STAT1*, and *IRF* showed significantly decreased expressed after receptor knockout. The results show that ISGs are activated through the JAK–STAT pathway under interferon stimulation but cannot be activated after receptor knockout. Thirdly, interferons have an effect in promoting the expression of ERV elements. After receptor knockout, the expression of ERV elements decreases under interferon stimulation. Some studies confirmed that certain ERVs possess ISRE or GAS elements that induce the expression of downstream ISGs [14]. This may be the reason for the change in the expression of some ERV elements after interferon stimulation. Finally, we investigated the gene regulatory networks related to HERVs that are induced by the proinflammatory cytokine IFN-β. In the PPI core network, *STAT1*, *STAT2*, and *IRF1* play key regulatory roles, with 54, 38, and 42 linked genes, respectively. These genes are also closely related to HERVs in DEHERV-G pairs, which is consistent with the findings of the mammalian genome ERV elements providing enhancer activity through directly binding with STAT1, IRF1, and NF-κB [18,46].

The ERV LTRs possess a series of transcription factor binding sites (TFBSs) involved in regulating the duration and strength of immune responses, including GATA factors (binding to GATA DNA sequences) [47], activator protein 1 (AP-1) and AP-2 [48,49], IRF1, IRF2, IRF17 [50], STAT [51], etc., the major players influencing the innate immune response. Combining our findings with those from research on ERVs, we assume that ERV elements and ISGs may share common TBFSs in regulating the host innate immune system [46,52,53]. However, our hypothesis is currently based on the results of bioinformatics analysis, and follow-up experiments are needed to provide evidence of a direct relationship between ERV elements and ISGs in these DEHERV-G pairs.

## 5. Conclusions

In summary, we analyzed the expression profiles of ERVs and human genes stimulated in THP1 cell lines after IFNAR knockout. Our results suggest that RNA expression of ISGs and ERV elements is correlated, indicating that ERV elements are closely associated with host innate immune responses.

## Data Availability

The data in this publication have been deposited in the NCBI Gene Expression Omnibus and are accessible through GEO Series accession number GSE211502.

## Supplementary Materials

The following supporting information can be downloaded at: https://www.mdpi.com/article/10.3390/v15010060/s1, Figure S1: Screening and classification of DEHERVs; Figure S2: Screening and classification of differentially expressed genes (DEGs); Table S1: The clean reads of HERVs and genes; Table S2: Common differentially expressed DEHERV-G pairs in the three groups; Table S3: Genes in the heat map; Table S4: The PPI network by MCODE algorithm.

## Abbreviations

The following abbreviations are used in this manuscript:

ERVs	endogenous retroviruses
REs	retrotransposon
TEs	transposable elements
LTRs	long terminal repeats
dsRNA	double-stranded RNA
TLR3	toll-like receptor 3
RIG-I	retinoic-acid-inducible gene I
MDA5	melanoma differentiation-associated gene 5
IFNAR	type I interferon receptor
JAK	Janus-activated kinase
STAT2	signal transducer and activator of transcription 2
IRF9	IFN regulatory factor 9
ISG	IFN-stimulated gene
ISGF3	IFN-stimulated gene factor 3
ISRE	interferon-stimulated regulatory element
GAS	IFN-γ-activated site
IRF1	interferon regulatory factor 1
KO	knockout
DAVID	Database for Annotation, Visualization, and Integrated Discovery
GO	Gene Ontology
KEGG	Kyoto Encyclopedia of Genes and Genomes
PPI	Protein–protein interaction
DE	differentially expressed
STRING	Search Tool for the Retrieval of Interacting Genes
PCA	principal component analysis
BP(s)	biological process(es)
CC	cell component
MF	molecular function
TFBSs	transcription factors binding sites
AP	activator protein

## References

[1] Bannert N., Kurth R. (2004). Retroelements and the human genome: New perspectives on an old relation. Proc. Natl. Acad. Sci. USA.

[2] Boller K., Schönfeld K., Lischer S., Fischer N., Hoffmann A., Kurth R., Tönjes R.R. (2008). Human endogenous retrovirus HERV-K113 is capable of producing intact viral particles. J. Gen. Virol..

[3] Gogvadze E., Buzdin A. (2009). Retroelements and their impact on genome evolution and functioning. Cell. Mol. Life Sci..

[4] Lander E.S., Linton L.M., Birren B., Nusbaum C., Zody M.C., Baldwin J., Devon K., Dewar K., Doyle M., FitzHugh W. (2001). Initial sequencing and analysis of the human genome. Nature.

[5] de Weerd N.A., Vivian J.P., Nguyen T.K., Mangan N.E., Gould J.A., Braniff S.J., Zaker-Tabrizi L., Fung K.Y., Forster S.C., Beddoe T. (2013). Structural basis of a unique interferon-β signaling axis mediated via the receptor IFNAR1. Nat. Immunol..

[6] Bannert N., Kurth R. (2006). The evolutionary dynamics of human endogenous retroviral families. Annu. Rev. Genom. Hum. Genet..

[7] Xue B., Zeng T., Jia L., Yang D., Lin S.L., Sechi L.A., Kelvin D.J. (2020). Identification of the distribution of human endogenous retroviruses K (HML-2) by PCR-based target enrichment sequencing. Retrovirology.

[8] Wolf G., Yang P., Füchtbauer A.C., Füchtbauer E.M., Silva A.M., Park C., Wu W., Nielsen A.L., Pedersen F.S., Macfarlan T.S. (2015). The KRAB zinc finger protein ZFP809 is required to initiate epigenetic silencing of endogenous retroviruses. Genes Dev..

[9] Ohtani H., Liu M., Zhou W., Liang G., Jones P.A. (2018). Switching roles for DNA and histone methylation depend on evolutionary ages of human endogenous retroviruses. Genome Res..

[10] Imbeault M., Helleboid P.Y., Trono D. (2017). KRAB zinc-finger proteins contribute to the evolution of gene regulatory networks. Nature.

[11] Bowie A.G., Unterholzner L. (2008). Viral evasion and subversion of pattern-recognition receptor signalling. Nat. Rev. Immunol..

[12] Chow J., Franz K.M., Kagan J.C. (2015). PRRs are watching you: Localization of innate sensing and signaling regulators. Virology.

[13] Zhou X., Singh M., Sanz Santos G., Guerlavais V., Carvajal L.A., Aivado M., Zhan Y., Oliveira M.M.S., Westerberg L.S., Annis D.A. (2021). Pharmacologic Activation of p53 Triggers Viral Mimicry Response Thereby Abolishing Tumor Immune Evasion and Promoting Antitumor Immunity. Cancer Discov..

[14] Platanias L.C. (2005). Mechanisms of type-I- and type-II-interferon-mediated signalling. Nat. Rev. Immunol..

[15] Darnell J.E., Kerr I.M., Stark G.R. (1994). Jak-STAT pathways and transcriptional activation in response to IFNs and other extracellular signaling proteins. Science.

[16] Jacques P., Jeyakani J., Bourque G. (2013). The majority of primate-specific regulatory sequences are derived from transposable elements. PLoS Genet..

[17] Ye M., Goudot C., Hoyler T., Lemoine B., Amigorena S., Zueva E. (2020). Specific subfamilies of transposable elements contribute to different domains of T lymphocyte enhancers. Proc. Natl. Acad. Sci. USA.

[18] Chuong E.B., Elde N.C., Feschotte C. (2016). Regulatory evolution of innate immunity through co-option of endogenous retroviruses. Science.

[19] Schmid C.D., Bucher P. (2010). MER41 repeat sequences contain inducible STAT1 binding sites. PLoS ONE.

[20] Piehler J., Thomas C., Garcia K.C., Schreiber G. (2012). Structural and dynamic determinants of type I interferon receptor assembly and their functional interpretation. Immunol. Rev..

[21] Harari D., Abramovich R., Zozulya A., Smith P., Pouly S., Köster M., Hauser H., Schreiber G. (2014). Bridging the species divide: Transgenic mice humanized for type-I interferon response. PLoS ONE.

[22] Urin V., Shemesh M., Schreiber G. (2019). CRISPR/Cas9-based Knockout Strategy Elucidates Components Essential for Type 1 Interferon Signaling in Human HeLa Cells. J. Mol. Biol..

[23] Edgar R., Domrachev M., Lash A.E. (2002). Gene Expression Omnibus: NCBI gene expression and hybridization array data repository. Nucleic Acids Res..

[24] Barrett T., Wilhite S.E., Ledoux P., Evangelista C., Kim I.F., Tomashevsky M., Marshall K.A., Phillippy K.H., Sherman P.M., Holko M. (2013). NCBI GEO: Archive for functional genomics data sets—Update. Nucleic Acids Res..

[25] Sanjana N.E., Shalem O., Zhang F. (2014). Improved vectors and genome-wide libraries for CRISPR screening. Nat. Methods.

[26] Shalem O., Sanjana N.E., Hartenian E., Shi X., Scott D.A., Mikkelson T., Heckl D., Ebert B.L., Root D.E., Doench J.G. (2014). Genome-scale CRISPR-Cas9 knockout screening in human cells. Science.

[27] Zhang L., Ma J., Jin X., Zhang L., Zhang M., Li P.Z., Li J., Zhang L. (2022). Human IFNAR2 mutant generated by CRISPR/Cas9-induced exon skipping upregulates a subset of tonic-like ISGs upon IFNβ stimulation. J. Interferon Cytokine Res..

[28] Roser L.G., Agüero F., Sánchez D.O. (2019). FastqCleaner: An interactive Bioconductor application for quality-control, filtering and trimming of FASTQ files. BMC Bioinf..

[29] Bolger A.M., Lohse M., Usadel B. (2014). Trimmomatic: A flexible trimmer for Illumina sequence data. Bioinformatics.

[30] Kim D., Langmead B., Salzberg S.L. (2015). HISAT: A fast spliced aligner with low memory requirements. Nat. Methods.

[31] Liao Y., Smyth G.K., Shi W. (2014). featureCounts: An efficient general purpose program for assigning sequence reads to genomic features. Bioinformatics.

[32] Paces J., Pavlícek A., Zika R., Kapitonov V.V., Jurka J., Paces V. (2004). HERVd: The Human Endogenous RetroViruses Database: Update. Nucleic Acids Res..

[33] Love M.I., Huber W., Anders S. (2014). Moderated estimation of fold change and dispersion for RNA-seq data with DESeq2. Genome Biol..

[34] Haase K., Mösch A., Frishman D. (2015). Differential expression analysis of human endogenous retroviruses based on ENCODE RNA-seq data. BMC Med. Genom..

[35] Huang D.W., Sherman B.T., Tan Q., Collins J.R., Alvord W.G., Roayaei J., Stephens R., Baseler M.W., Lane H.C., Lempicki R.A. (2007). The DAVID Gene Functional Classification Tool: A novel biological module-centric algorithm to functionally analyze large gene lists. Genome Biol..

[36] Ashburner M., Ball C.A., Blake J.A., Botstein D., Butler H., Cherry J.M., Davis A.P., Dolinski K., Dwight S.S., Eppig J.T. (2000). Gene ontology: Tool for the unification of biology. The Gene Ontology Consortium. Nat. Genet..

[37] Kanehisa M. (2002). The KEGG database. Novartis Found. Symp..

[38] Franceschini A., Szklarczyk D., Frankild S., Kuhn M., Simonovic M., Roth A., Lin J., Minguez P., Bork P., von Mering C. (2013). STRING v9.1: Protein-protein interaction networks, with increased coverage and integration. Nucleic Acids Res..

[39] Smoot M.E., Ono K., Ruscheinski J., Wang P.L., Ideker T. (2011). Cytoscape 2.8: New features for data integration and network visualization. Bioinformatics.

[40] Wheeler T.J., Clements J., Eddy S.R., Hubley R., Jones T.A., Jurka J., Smit A.F., Finn R.D. (2013). Dfam: A database of repetitive DNA based on profile hidden Markov models. Nucleic Acids Res..

[41] Wang M., Qiu Y., Liu H., Liang B., Fan B., Zhou X., Liu D. (2020). Transcription profile of human endogenous retroviruses in response to dengue virus serotype 2 infection. Virology.

[42] Liu S.Y., Sanchez D.J., Aliyari R., Lu S., Cheng G. (2012). Systematic identification of type I and type II interferon-induced antiviral factors. Proc. Natl. Acad. Sci. USA.

[43] Indraccolo S., Pfeffer U., Minuzzo S., Esposito G., Roni V., Mandruzzato S., Ferrari N., Anfosso L., Dell’Eva R., Noonan D.M. (2007). Identification of genes selectively regulated by IFNs in endothelial cells. J. Immunol..

[44] Salamon D., Adori M., He M., Bönelt P., Severinson E., Kis L.L., Wu L., Ujvari D., Leveau B., Nagy N. (2012). Type I interferons directly down-regulate BCL-6 in primary and transformed germinal center B cells: Differential regulation in B cell lines derived from endemic or sporadic Burkitt’s lymphoma. Cytokine.

[45] Zhang X., Yang W., Wang X., Zhang X., Tian H., Deng H., Zhang L., Gao G. (2018). Identification of new type I interferon-stimulated genes and investigation of their involvement in IFN-β activation. Protein Cell.

[46] Manghera M., Ferguson-Parry J., Lin R., Douville R.N. (2016). NF-κB and IRF1 Induce Endogenous Retrovirus K Expression via Interferon-Stimulated Response Elements in Its 5’ Long Terminal Repeat. J. Virol..

[47] Wang L., Wang X., Adamo M.L. (2000). Two putative GATA motifs in the proximal exon 1 promoter of the rat insulin-like growth factor I gene regulate basal promoter activity. Endocrinology.

[48] Nead M.A., Baglia L.A., Antinore M.J., Ludlow J.W., McCance D.J. (1998). Rb binds c-Jun and activates transcription. EMBO J..

[49] Imhof A., Schuierer M., Werner O., Moser M., Roth C., Bauer R., Buettner R. (1999). Transcriptional regulation of the AP-2alpha promoter by BTEB-1 and AP-2rep, a novel wt-1/egr-related zinc finger repressor. Mol. Cell. Biol..

[50] Arany I., Grattendick K.J., Whitehead W.E., Ember I.A., Tyring S.K. (2003). A functional interferon regulatory factor-1 (IRF-1)-binding site in the upstream regulatory region (URR) of human papillomavirus type 16. Virology.

[51] Soldaini E., John S., Moro S., Bollenbacher J., Schindler U., Leonard W.J. (2000). DNA binding site selection of dimeric and tetrameric Stat5 proteins reveals a large repertoire of divergent tetrameric Stat5a binding sites. Mol. Cell. Biol..

[52] Cheon H., Holvey-Bates E.G., Schoggins J.W., Forster S., Hertzog P., Imanaka N., Rice C.M., Jackson M.W., Junk D.J., Stark G.R. (2013). IFNβ-dependent increases in STAT1, STAT2, and IRF9 mediate resistance to viruses and DNA damage. EMBO J..

[53] Kassiotis G., Stoye J.P. (2016). Immune responses to endogenous retroelements: Taking the bad with the good. Nat. Rev. Immunol..

