# Supplementary material for: Endogenous Retrovirus Elements Are Co-Expressed with IFN Stimulation Genes in the JAK–STAT Pathway
Source: Viruses. 2022 Dec 24;15(1):60. doi: 10.3390/v15010060 (PMC9861321; doi:10.3390/v15010060)
Supplement: Supplementary file 1 [file viruses-15-00060-s001.zip › Supplementary Figure S1-2.pdf]

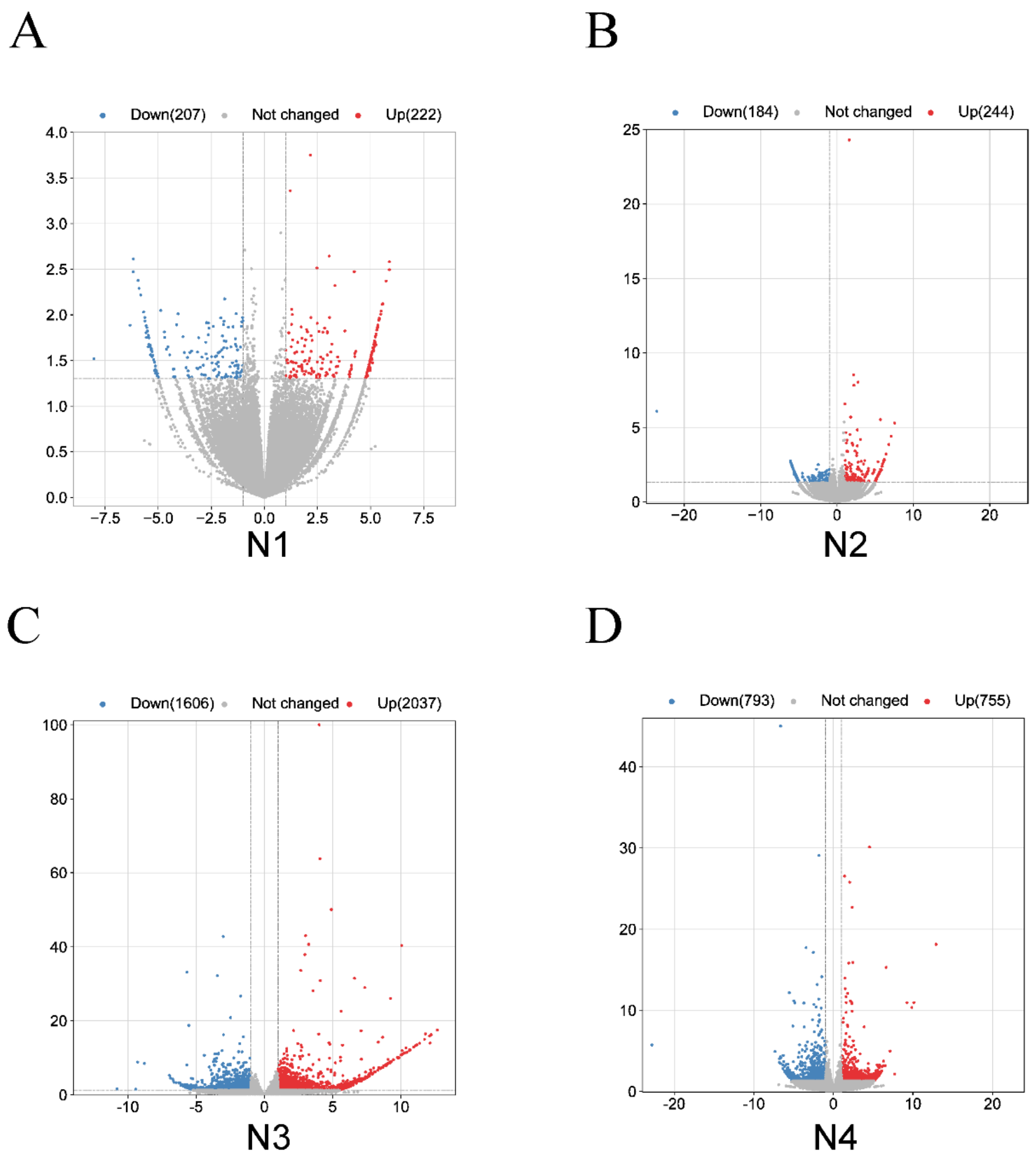

Supplementary Figure S1. Screening and classification of DEHERVs. (A) Volcano plot of group N1 (THP1_IFNAR1_KO cell lines under interferon stimulation vs THP1_IFNAR1_KO cell lines without interferon stimulation). (B) Volcano plot of group N2 (THP1_IFNAR2_mutant cell lines under interferon stimulation vs THP1_IFNAR2_mutant cell lines without interferon stimulation). (C) Volcano plot of group N3 (THP1 without interferon stimulation vs THP1_IFNAR1_KO cell lines without interferon stimulation). (D) Volcano plot of group N4 (THP1 without interferon stimulation vs THP1_IFNAR2_mutant cell lines without interferon stimulation). Dots with red color and blue color represent upregulated and downregulated HERV loci, respectively (adjusted P-value < 0.05 and $|\log2 \text{ fold change}| > 1$). Dots with gray color represent loci with no significant differential expression.

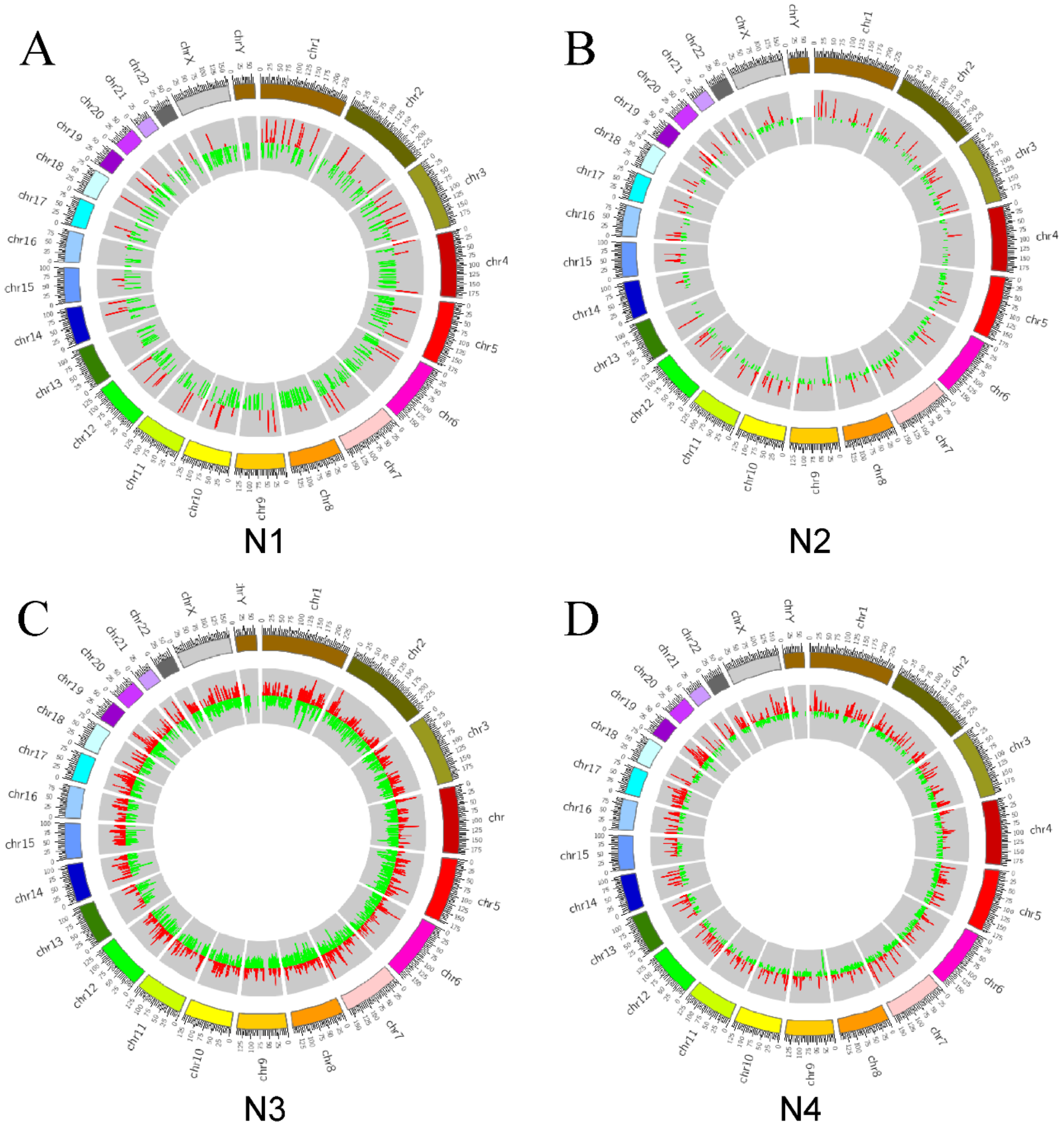

Supplementary Figure S2. Screening and classification of differentially expressed genes (DEGs). Circos plot showing DEGs (red column) and DEHERVs (green column) across chromosomal locations. (A) Circos plot of group N1 (THP1_IFNAR1_KO cell lines under interferon stimulation vs THP1_IFNAR1_KO cell lines without interferon stimulation). (B) Circos plot of group N2 (THP1_IFNAR2_mutant cell lines under interferon stimulation vs THP1_IFNAR2_mutant cell lines without interferon stimulation). (C) Circos plot of group N3 (THP1 without interferon stimulation vs THP1_IFNAR1_KO cell lines without interferon stimulation). (D) Circos plot of group N4 (THP1 without interferon stimulation vs THP1_IFNAR2_mutant cell lines without interferon stimulation). The length of the column represents the absolute value of the log2 fold change.
