# Supplementary figures and images for: Endogenous Retrovirus Elements Are Co-Expressed with IFN Stimulation Genes in the JAK–STAT Pathway
Source: Viruses. 2022 Dec 24;15(1):60. doi: 10.3390/v15010060 (PMC9861321; doi:10.3390/v15010060)

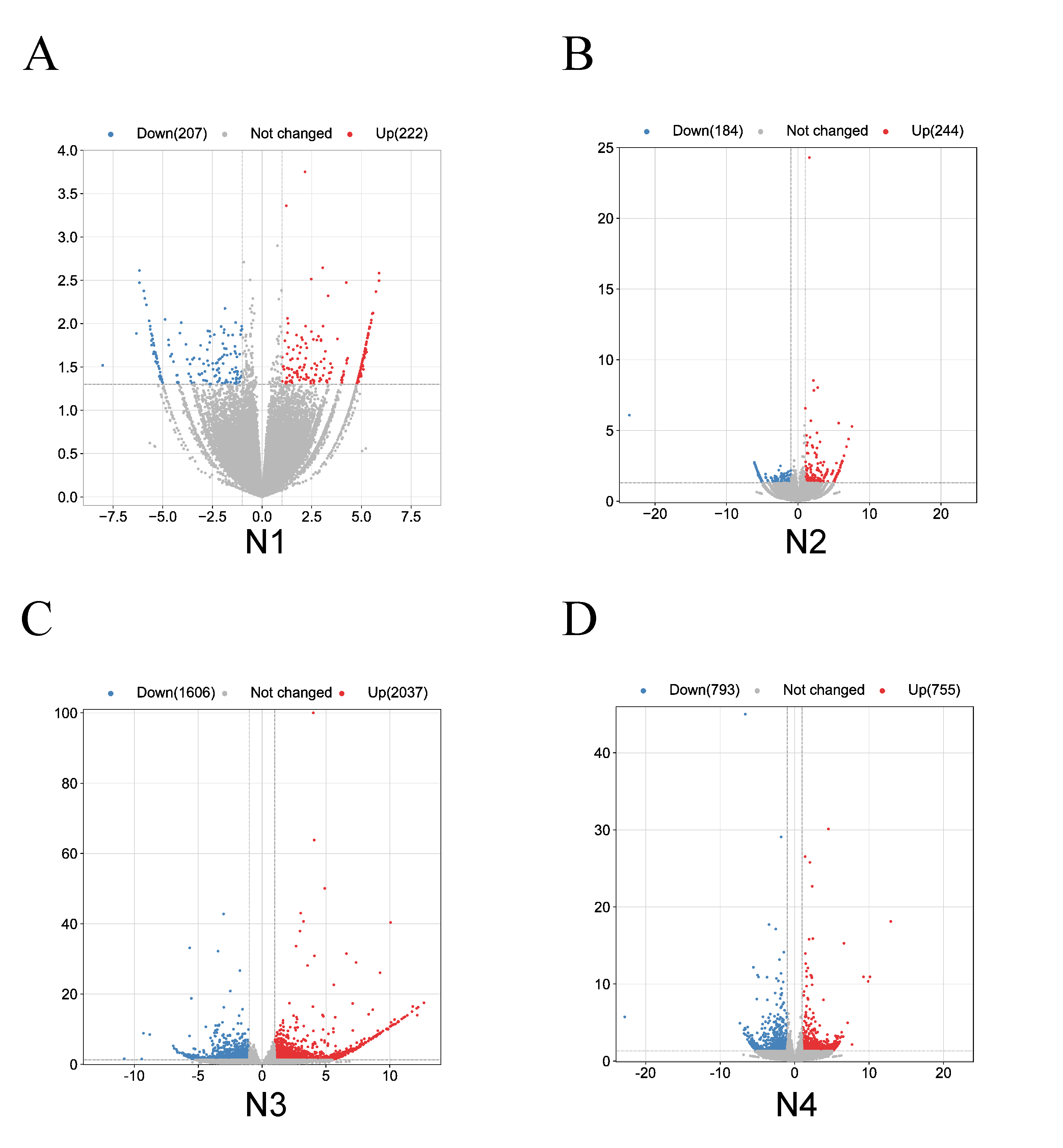

Supplement: Supplementary file 1 [file viruses-15-00060-s001.zip › Figure S1.tif]

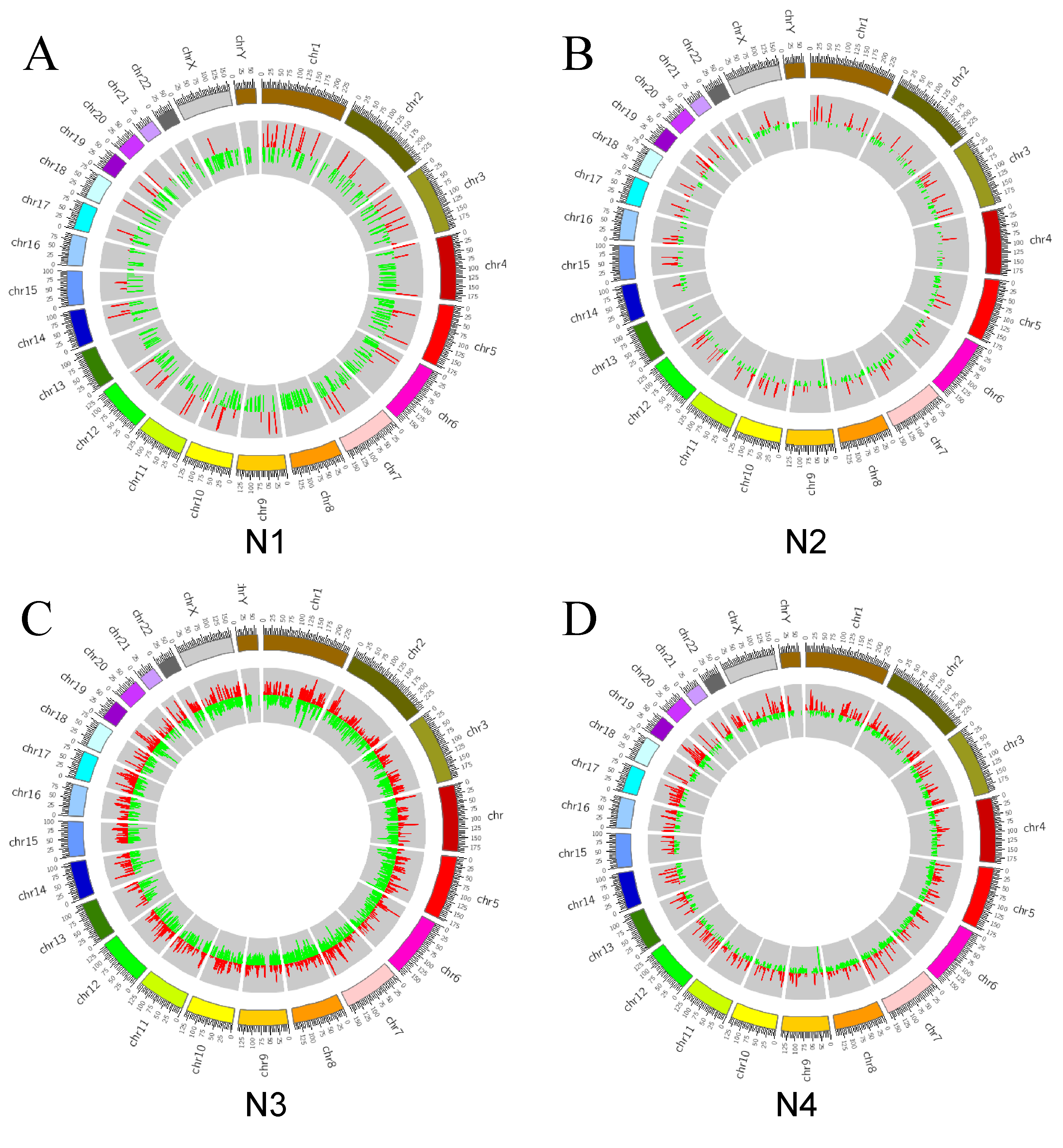

Supplement: Supplementary file 1 [file viruses-15-00060-s001.zip › Figure S2.tif]
